# Simultaneous Visualization of ^161^Tb- and ^177^Lu-Labeled Somatostatin Analogues Using Dual-Isotope SPECT Imaging

**DOI:** 10.3390/pharmaceutics13040536

**Published:** 2021-04-12

**Authors:** Francesca Borgna, Patrick Barritt, Pascal V. Grundler, Zeynep Talip, Susan Cohrs, Jan Rijn Zeevaart, Ulli Köster, Roger Schibli, Nicholas P. van der Meulen, Cristina Müller

**Affiliations:** 1 Center for Radiopharmaceutical Sciences, Paul Scherrer Institute, 5232 Villigen-PSI, Switzerland; francesca.borgna@psi.ch (F.B.); pabarritt@me.com (P.B.); pascal.grundler@psi.ch (P.V.G.); zeynep.talip@psi.ch (Z.T.); susan.cohrs@psi.ch (S.C.); roger.schibli@psi.ch (R.S.); nick.vandermeulen@psi.ch (N.P.v.d.M.); 2 Radiochemistry, South African Nuclear Energy Corporation (Necsa), Brits 0240, South Africa; janrijn.zeevaart@necsa.co.za; 3 Institut Laue-Langevin, 38042 Grenoble, France; koester@ill.fr; 4 Department of Chemistry and Applied Biosciences, ETH Zurich, 8093 Zurich, Switzerland; 5 Laboratory of Radiochemistry, Paul Scherrer Institute, 5232 Villigen-PSI, Switzerland

**Keywords:** terbium-161, lutetium-177, NET, antagonists, agonists, radionuclide therapy, dual-isotope SPECT

## Abstract

The decay of terbium-161 results in the emission of β¯-particles as well as conversion and Auger electrons, which makes terbium-161 interesting for therapeutic purposes. The aim of this study was to use dual-isotope SPECT imaging in order to demonstrate visually that terbium-161 and lutetium-177 are interchangeable without compromising the pharmacokinetic profile of the radiopharmaceutical. The ^161^Tb- and ^177^Lu-labeled somatostatin (SST) analogues DOTATOC (agonist) and DOTA-LM3 (antagonist) were tested in vitro to demonstrate equal properties regarding distribution coefficients and cell uptake into SST receptor-positive AR42J tumor cells. The radiopeptides were further investigated in AR42J tumor-bearing nude mice using the method of dual-isotope (terbium-161/lutetium-177) SPECT/CT imaging to enable the visualization of their distribution profiles in the same animal. Equal pharmacokinetic profiles were demonstrated for either of the two peptides, irrespective of whether it was labeled with terbium-161 or lutetium-177. Moreover, the visualization of the sub-organ distribution confirmed similar behavior of ^161^Tb- and ^177^Lu-labeled SST analogues. The data were verified in quantitative biodistribution studies using either type of peptide labeled with terbium-161 or lutetium-177. While the radionuclide did not have an impact on the organ distribution, this study confirmed previous data of a considerably higher tumor uptake of radiolabeled DOTA-LM3 as compared to the radiolabeled DOTATOC.

## 1. Introduction

Terbium-161 was previously proposed as an alternative to lutetium-177 for tumor-targeted radionuclide therapy [1,2]. It is a β¯-particle emitter with similar decay properties (T_1/2_ = 6.95 d [3]; Eβ¯_average_ = 154 keV) to those of lutetium-177 (T_1/2_ = 6.65 d; Eβ¯_average_ = 134 keV); however, terbium-161 also emits conversion and Auger electrons that are characterized by a high linear energy transfer (LET). Theoretical dose calculations revealed a significantly increased absorbed dose to small spheres when using terbium-161 as compared to lutetium-177 [4,5,6,7,8]. ^161^Tb-labeled radiopharmaceuticals are, thus, believed to be parti-cularly effective for the treatment of disseminated disease, possibly enabling the killing of even single cancer cells. In analogy to lutetium-177 that emits γ-radiation (Eγ = 112.9 keV, I = 6.2% and 208.4 keV, I = 10%) useful for single photon emission computed tomo-graphy (SPECT) imaging, the decay of terbium-161 also results in the emission of photons (Eγ = 48.9 keV, I = 17% and 74.6 keV, I = 10%) that enables imaging using SPECT [9,10,11,12].

Due to the chemical similarity of terbium-161 and lutetium-177, which both belong to the group of lanthanides, the coordination of terbium-161 can be readily achieved using a 1,4,7,10-tetraazacyclododecane-1,4,7,10-tetraacetic acid (DOTA)-chelator as demonstrated in preclinical experiments with small molecule-based targeting agents, such as DOTA-folate conjugates and PSMA-617 [9,10,13]. Although it was previously shown that peptides such as somatostatin (SST) analogues are more sensitive to the exchange of the radiometal than small molecules [14,15,16], it was assumed that the biodistribution of SST analogues would be equal, irrespective of whether terbium or lutetium radionuclides were employed [17,18].

It is believed that terbium-161 would be particularly interesting in combination with SST analogues for the treatment of neuroendocrine and other somatostatin receptor (SSTR)-positive tumors. Radiolabeled SSTR-targeting agonists (e.g., DOTATOC [19]) and antagonists (e.g., DOTA-LM3 [16,20]) have been clinically employed for radiotheragnostic purposes (Supplementary Materials, Figure S1) [21,22,23,24]. The aim of this study was to compare the in vitro and in vivo characteristics of DOTATOC and DOTA-LM3 after labeling with terbium-161 and lutetium-177. The method of dual-isotope SPECT/CT imaging was used for direct comparison of the ^161^Tb- and ^177^Lu-labeled versions of these peptides, as well as their sub-organ distribution pattern in the same animal.

## 2. Materials and Methods

### 2.1. Terbium-161 and Lutetium-177

Terbium-161 was produced using the ^160^Gd(n,γ)^161^Gd→^161^Tb nuclear reaction by neutron irradiation of enriched gadolinium-160 targets (98.2%, Isoflex, San Francisco, CA, USA) at the SAFARI-1 reactor at Necsa, Pelindaba, South Africa, or at the RHF reactor at Institut Laue Langevin, Grenoble, France. After chemical separation, no-carrier-added (n.c.a.) [^161^Tb]TbCl_3_ was obtained in 0.05 M HCl, as previously reported [25]. Lutetium-177 was obtained as n.c.a. [^177^Lu]LuCl_3_ in 0.04 M HCl from ITM Medical Isotopes GmbH, Munich, Germany.

### 2.2. Preparation and In Vitro Evaluation of the Radiopeptides

DOTA-[Tyr^3^]-octreotide (DOTATOC) was kindly provided by ITM Medical Isotopes GmbH, Munich, Germany. DOTA-LM3 was purchased as a custom synthesis from CSBio (Silicon Valley Menlo Park, CA, USA). ^161^Tb- and ^177^Lu-labeled DOTATOC and DOTA-LM3 were prepared under standard labeling conditions, as previously reported for other biomolecules (Supplementary Materials) [9,13]. The radiolytic stability of the radiopeptides (40 MBq/mL) in the absence and presence of l-ascorbic acid (120 μg/10 MBq), as well as their *n*-octanol/PBS distribution coefficients (logD values), were determined as previously reported (Supplementary Materials) [26].

### 2.3. Cell Culture

AR42J tumor cells (SSTR-positive exocrine rat pancreatic cancer cells, ECACC 93100618) [27] were purchased from Health Protection Agency Culture Collections (Salisbury, UK). The cells were cultured in Roswell Park Memorial Institute (RPMI) medium supplemented with glutamine, antibiotics, and 20% fetal calf serum.

### 2.4. In Vitro Studies

Cell uptake and internalization studies were performed after seeding AR42J tumor cells (10^6^ cells/2 mL) in poly-l-lysine-coated 12-well-plates in RPMI medium with supplements and incubation (37 °C, 5% CO_2_) overnight. After washing the cells with phosphate buffered saline (PBS) pH 7.4, assay medium (975 μL; RPMI supplemented with glutamine, antibiotics and 1% fetal calf serum) and 25 μL radiopeptide solution (~15 kBq, ~0.75 pmol per well) were added to each well, resulting in a radioligand concentration of 0.75 nM. The well-plates were incubated under standard cell culture conditions (37 °C, 5% CO_2_) for 0.5 h, 2 h or 4 h. SSTR-blocking experiments were performed using 1 μM DOTATOC or 1 μM DOTA-LM3, respectively (Supplementary Materials). To determine the total cell uptake, the supernatants were removed and the cells washed three times with ice-cold PBS. The internalized fraction was determined by stripping the cells with a glycine-based acidic buffer (pH 2.8) for 30 min, followed by two washing steps with ice-cold PBS. Cell samples were lysed by addition of NaOH (1 M, 1 mL) to each well. The radioactive cell lysates were measured in a γ-counter (Wallac Wizard 1480, Perkin Elmer Waltham, MA, USA). The activity of the samples was standardized to the average protein concentration in each well (~0.3 mg) using a Micro BCA Protein Assay kit (Pierce, Thermo Scientific, Waltham, MA, USA). Experiments were performed three times, in triplicate.

Saturation binding assays were performed to determine the K_D_ values. AR42J cells, grown in 12-well plates, were washed with PBS pH 7.4, before the addition of assay medium or assay medium containing 1 μM DOTANOC or DOTA-LM3, respectively, followed by incubation for 30 min at 37 °C. Variable concentrations of the respective radiopeptide were added to the cells (25 μL, 0.1–1000 nM, 20 MBq/nmol) before incubation for 1 h under standard cell culture conditions. The total uptake of activity in cells of each well was determined after two washing steps using PBS (pH 7.4) followed by cell lysis in a solution of NaOH (1 M, 1 mL) and measurement with a γ-counter. The specific binding was calculated as the difference between measured activity in the bound fraction and the fraction co-exposed to the unlabeled peptide, to determine unspecific binding of the radiopeptides. K_D_ values were calculated using GraphPad Prism software (version 8.0, Graph Pad software, San Diego, CA, USA). The final K_D_ value was determined as the average of three independent experiments performed in triplicate.

### 2.5. In Vivo Studies

All applicable international, national, and/or institutional guidelines for the care and use of laboratory animals, in particular, the guidelines of Swiss Regulations for Animal Welfare were applied. The preclinical studies were ethically approved by the responsible Committee of Animal Experimentation and permitted by the responsible cantonal authorities (license No. 75721; approval date: 25 September 2018). Five-week-old female CD-1 nude mice were purchased from Charles River Laboratories (Sulzfeld, Germany). Mice were subcutaneously inoculated with AR42J tumor cells (5 × 10^6^ cells in 100 µL PBS) for biodistribution (*n* = 3–4 mice per timepoint) and SPECT/CT imaging studies (*n* = 2–3 mice per setting). The studies were performed 10–14 days after tumor cell inoculation when the tumor size reached a volume of ~250 mm^3^.

### 2.6. Biodistribution Studies

Biodistribution studies were performed after intravenous (i.v.) injection of the mice with [^161^Tb]Tb-DOTATOC or [^177^Lu]Lu-DOTATOC (5 MBq; 1 nmol in 100 µL/mouse) diluted in PBS containing 0.05% bovine serum albumin. [^161^Tb]Tb-DOTA-LM3 or [^177^Lu]Lu-DOTA-LM3 were used under the same conditions. The mice were sacrificed at 2 h and 24 h post injection (p.i.). Selected tissues and organs (blood, heart, lung, spleen, kidneys, adrenals, stomach, pancreas, intestines, liver, femoral muscle, femur, and tumor) were collected, weighed, and the accumulated activity was counted in a γ-counter. The decay-corrected data were listed as the percentage of the injected activity per gram of tissue mass (% IA/g).

### 2.7. Dual-Isotope SPECT/CT Imaging Studies

SPECT/CT scans were performed with a dedicated small-animal SPECT/CT scanner (NanoSPECT/CT, Mediso Medical Imaging Systems, Budapest, Hungary; Supplementary Materials). The scans were acquired using Nucline software (version 1.02, Mediso Ltd., Budapest, Hungary). Simultaneous acquisition of counts stemming from terbium-161 and lutetium-177, respectively, was performed by the selection of distinct energy windows for the two radionuclides. The two energy windows chosen for terbium-161 were set at 47.7 keV ± 10%, which enabled the detection of X-rays and γ-rays (46.0 keV, 48.9 keV and 52.0 keV), and at 74.6 keV ± 10%, enabling the detection of the γ-rays at 74.6 keV. For lutetium-177, the windows were set at 112.9 keV ± 10% and 208.4 ± 10% to detect the γ-rays at 112.9 keV and 208.4 keV, respectively. SPECT data were reconstructed iteratively using HiSPECT software (version 1.4.3049, Scivis GmbH, Göttingen, Germany). The CT was reconstructed in real time using a cone-beam filtered backprojection. The fused datasets of SPECT and CT scans were analyzed using the VivoQuant postprocessing software (version 3.5, inviCRO Imaging Services and Software, Boston, MA, USA). A Gaussian post-reconstruction filter (FWHM = 1.0 mm) was applied. Images were prepared using CorelDRAW (version X7, Corel Corporation, Ottawa, Ontario, Canada).

The established dual-isotope SPECT imaging protocol was evaluated with regard to a potential interference of the photon emission of terbium-161 and lutetium-177, respectively (Supplementary Materials).

Mice were i.v. injected with a mixture of [^161^Tb]Tb-DOTATOC (~15 MBq, 0.5 nmol/mouse) and [^177^Lu]Lu-DOTATOC (~15 MBq, 0.5 nmol/mouse) or a mixture of [^161^Tb]Tb-DOTA-LM3 and [^177^Lu]Lu-DOTA-LM3 in PBS containing 0.05% bovine serum albumin and ascorbic acid (~300 μg/30 MBq). Blocking studies were performed under the same experimental conditions; however, in this case, an excess (20 nmol/mouse) of unlabeled DOTATOC or DOTA-LM3 was added to the injection solution. SPECT/CT scans were acquired 2 h, 4 h, and 24 h after injection of the radiopeptides using the dual-isotope SPECT acquisition protocol with a frame time of 60 s resulting in a scan time of 45 min. During the in vivo scans, mice were anesthetized by inhalation of a mixture of isoflurane and oxygen. Quantification of the accumulated activity in tumors and kidneys was performed using the quantification tool of the VivoQuant postprocessing software (version 3.5, inviCRO Imaging Services and Software, Boston, MA, USA) (Supplementary Materials).

### 2.8. Statistical Analysis

The in vitro data (logD value, percentage of cell uptake and K_D_ values) were analyzed for significance applying a one-way or two-way ANOVA with Tukey’s multiple comparisons post-test using GraphPad Prism software (version 8.0, Graph Pad software, San Diego, CA, USA). Biodistribution data were analyzed for statistical significance using a two-way ANOVA with Sidak’s multiple comparisons post-test. A *p* value ≤ 0.05 was considered as statistically significant.

## 3. Results and Discussion

### 3.1. Equal Labeling Conditions Can Be Applied for Terbium-161 and Lutetium-177

The product specifications of terbium-161 were comparable to that of n.c.a. lutetium-177, as previously reported by Gracheva et al. [25]. This enabled us to label the SST analogues at molar activities of up to 100 MBq/nmol with radiochemical purity of >98% (Supplementary Materials, Figure S2). This molar activity was higher than what would be required for GMP-produced radiopeptides for clinical application, which is commonly 5.55–7.4 GBq/200 μg (i.e., 39–53 MBq/nmol for [^177^Lu]Lu-DOTATOC) [28,29,30].

### 3.2. Radiolytic Stability of ^161^Tb- and ^177^Lu-Labeled SST Analogues Is Comparable

All radiopeptides, irrespective of whether labeled with terbium-161 or lutetium-177, were stable (>90% of intact radiopeptide) up to 1 h after preparation. The intact fraction was, however, reduced to ~90% and ~60% after 4 h and 24 h, respectively, due to radiolytic degradation. In line with published data [10,25], the percentage of intact ^161^Tb- and ^177^Lu-labeled counterparts was identical, indicating that potential formation of additional reactive oxygen species (ROS) due to short-ranged conversion and Auger electrons did not affect the radiopeptides’ integrity [31]. Stabilization of radiopeptides for clinical application is commonly achieved by the addition of ascorbic acid [32], which was also effective for the stabilization of the ^161^Tb- and ^177^Lu-labeled SST analogues over longer time periods (Supplementary Materials, Figure S3).

### 3.3. The In Vitro Properties of ^161^Tb- and ^177^Lu-Labeled Peptides Are Equal

**Figure 1:**
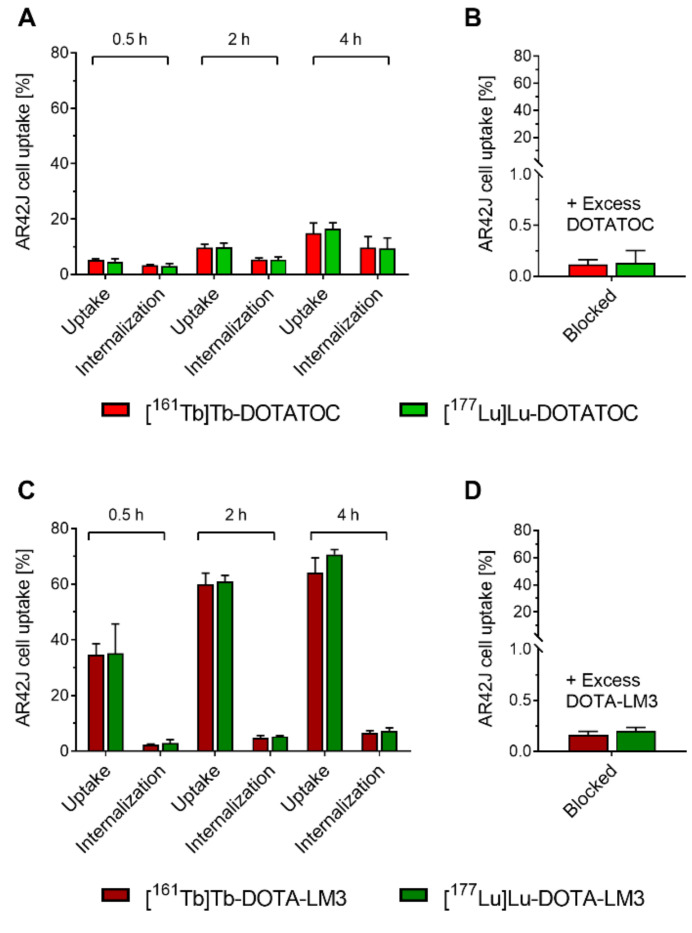
Results of the in vitro uptake and internalization of the radiopeptides in AR42J tumor cells after incubation for 0.5 h, 2 h and 4 h. (**A**) [^161^Tb]Tb-DOTATOC and [^177^Lu]Lu-DOTATOC; (**B**) [^161^Tb]Tb-DOTATOC and [^177^Lu]Lu-DOTATOC with excess DOTATOC; (**C**) [^161^Tb]Tb-DOTA-LM3 and [^177^Lu]Lu-DOTA-LM3; (**D**) [^161^Tb]Tb-DOTA-LM3 and [^177^Lu]Lu-DOTA-LM3 with excess DOTA-LM3.

As expected, the ^161^Tb- and ^177^Lu-labeled counterparts revealed similar logD values ([^161^Tb]Tb-/[^177^Lu]Lu-DOTATOC: −3.3 ± 0.2 and −3.1 ± 0.2, respectively, *p* > 0.05; [^161^Tb]Tb-/[^177^Lu]Lu-DOTA-LM3: −2.5 ± 0.1 and −2.5 ± 0.1, respectively; *p* > 0.05). The AR42J cell uptake and internalization were also equal for the respective ^161^Tb- and ^177^Lu-labeled SSTR analogues (*p* > 0.05) (Figure 1).

The uptake of [^161^Tb]Tb-/[^177^Lu]Lu-DOTATOC into AR42J cells was highest (~15% of total added activity) after 4 h of incubation. Acid-washed cells retained 40–50% of the total uptake, which indicated efficient internalization of the radiopeptide-receptor complex as previously reported [33]. [^161^Tb]Tb-/[^177^Lu]Lu-DOTA-LM3 showed higher AR42J cell uptake at all investigated timepoints and reached almost 70% after an incubation period of 4 h. The internalized fraction of radiolabeled DOTA-LM3 was, however, almost negligible (<10% of the total uptake) which is a characteristic feature of SSTR antagonists as previously demonstrated [20].

The addition of excess DOTATOC or DOTA-LM3 prevented the cell uptake of [^161^Tb]Tb-/[^177^Lu]Lu-DOTATOC and [^161^Tb]Tb-/[^177^Lu]Lu-DOTA-LM3, respectively, which confirmed SSTR-specific binding of the radiopeptides. The use of a SSTR-agonist, such as DOTANOC, was, however, not effective to block the uptake of radiolabeled DOTA-LM3 completely (Supplementary Materials, Figure S4), because SSTR-antagonists can access more binding sites on the cell membrane (up to 14-fold) compared to SSTR-agonists [34]. Possibly, this can be attributed to the capacity of antagonists to bind to a larger number of SSTR conformations [35].

The K_D_ values of the ^161^Tb- and ^177^Lu-labeled counterparts were not significantly different (*p* > 0.05), thus, confirming that the radiopeptides bound equally well to SSTRs regardless of whether the respective peptide (DOTATOC or DOTA-LM3) was labeled with terbium-161 or lutetium-177. The SSTR-bindingaffinities of [^161^Tb]Tb-DOTA-LM3 and [^177^Lu]Lu-DOTA-LM3 (K_D_ values: 2.1 ± 0.9 nM and 1.6 ± 0.3 nM, respectively) were somewhat higher than for [^161^Tb]Tb-DOTATOC and [^177^Lu]Lu-DOTATOC (K_D_ values: 7.5 ± 0.9 nM and 10 ± 3 nM, respectively). The SSTR affinity was, however, for both types of SST analogues in the low nanomolar range, which is in line with previously published data [16,36,37].

### 3.4. Terbium-161 Does Not Change the Biodistribution of the SST Analogues Otherwise Labeled with Lutetium-177

**Figure 2:**
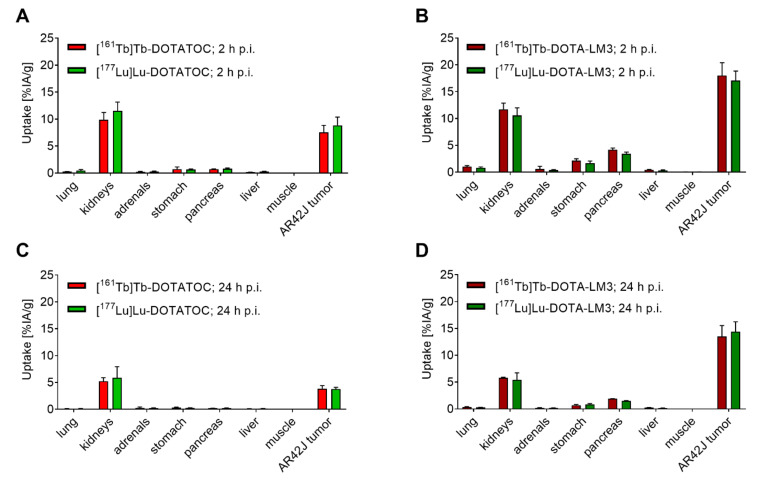
Biodistribution data obtained in AR42J tumor-bearing mice. (**A**,**B**) Tissue distribution at 2 h after injection of the radiopeptides; (**C**,**D**) Tissue distribution at 24 h after injection of the radiopeptides. The results are presented as percentage of injected activity per tissue mass (% IA/g). (**A**,**C**) Data obtained with [^161^Tb]Tb-DOTATOC and [^177^Lu]Lu-DOTATOC (*p* > 0.05); (**B**,**D**) Data obtained with [^161^Tb]Tb-DOTA-LM3 and [^177^Lu]Lu-DOTA-LM3 (*p* >0.05).

The biodistribution of ^161^Tb-labeled DOTATOC was comparable to that of ^177^Lu-labeled DOTATOC, demonstrated by similar activity accumulation in tissues and organs at both investigated timepoints (2 h and 24 h p.i.; *p* > 0.05). Equal tissue distribution was also observed when using DOTA-LM3, irrespective of whether it was labeled with terbium-161 or lutetium-177 (Figure 2 and Supplementary Materials, Tables S1 and S2).

These findings confirmed that terbium-161 and lutetium-177 are interchangeable without alteration of the radiopeptide’s pharmacokinetic properties, as already demonstrated with ^161^Tb- and ^177^Lu-labeled folate conjugates and PSMA ligands [9,10].

### 3.5. Variable Peptides (Agonist vs. Antagonist) Have Different Tissue Distribution Profiles

While our data showed that the choice of the radiolanthanide (terbium-161 vs. lutetium-177) did not have an impact on the tissue distribution of the radiopeptide, the type of SSTR-targeting peptide certainly did (Figure 2 and Supplementary Materials, Tables S1 and S2). As previously demonstrated, the difference between the uptake of the agonist and antagonist, respectively, was particularly obvious in SSTR-positive organs [38]. The radiolabeled DOTA-LM3 had a more than 2-fold higher tumor accumulation (~18% IA/g; 2 h p.i.) as compared to the radiolabeled DOTATOC (~9% of IA/g; 2 h p.i.; *p* <0.05). Moreover, the radiolabeled DOTA-LM3 was better retained in the tumor tissue (~14% IA/g; 24 h p.i.) than the radiolabeled DOTATOC (~4% IA/g, 24 h p.i.; *p* <0.05). The accumulation in other SSTR-positive organs such as lungs, adrenals, stomach and pancreas [39] was negligible in the case of radiolabeled DOTATOC (<1% IA/g; 2 h p.i. and <0.3% IA/g; 24 h p.i.), but higher (*p* <0.05) for radiolabeled DOTA-LM3 in the stomach and pancreas (~2% and ~4% IA/g at 2 h p.i., respectively). In the kidneys, the activity accumulation was low (~10% IA/g; 2 h p.i. and ~5.5% IA/g; 24 h p.i.), irrespective of the SST analogue (*p* > 0.05) while activity in other organs and tissues, including the blood, spleen and liver was negligible, in line with previously published data [20,40].

### 3.6. Simultaneous In Vivo Imaging Demonstrates Equal Biodistribution of ^161^Tb- and ^177^Lu-Labeled SST Analogues

The verification of the dual-isotope SPECT imaging protocol using Eppendorf vials filled with terbium-161 or lutetium-177 or both together confirmed the absence of significant cross talk between the radiolanthanides (Supplementary Materials, Figure S5).

**Figure 3:**
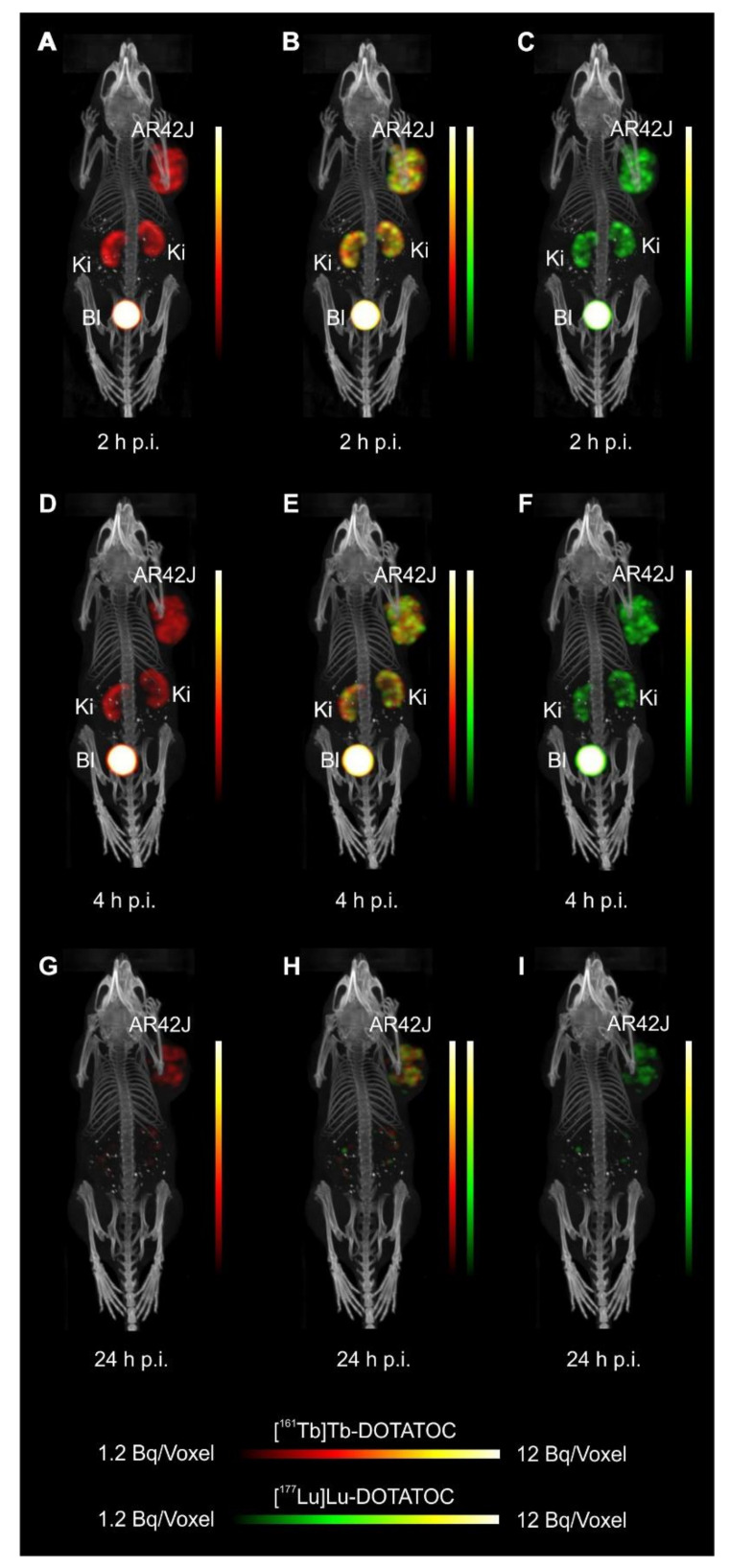
Dual-isotope SPECT/CT images of AR42J tumor-bearing mice shown as maximum intensity projections (MIPs) 2 h, 4 h and 24 h after injection of [^161^Tb]Tb-DOTATOC (15 MBq, 0.5 nmol/mouse) and [^177^Lu]Lu-DOTATOC (15 MBq, 0.5 nmol/mouse). (**A**–**C**) Scans acquired 2 h p.i. of the radiopeptides; (**D**–**F**) Scans acquired 4 h p.i. of the radiopeptides and (**G**–**I**) Scans acquired 24 h p.i. of the radiopeptides. (**A**,**D**,**G**) Reconstructions based on the X-rays and γ-lines of terbium-161; (**B**,**E**,**H**) Reconstructions based on the X-rays and γ-lines of terbium-161 and the γ-lines of lutetium-177; (**C**,**F**,**I**) Reconstructions based on the γ-lines of lutetium-177. AR42J = SSTR-positive tumor xenograft; Ki = kidney; Bl = urinary bladder.

**Figure 4:**
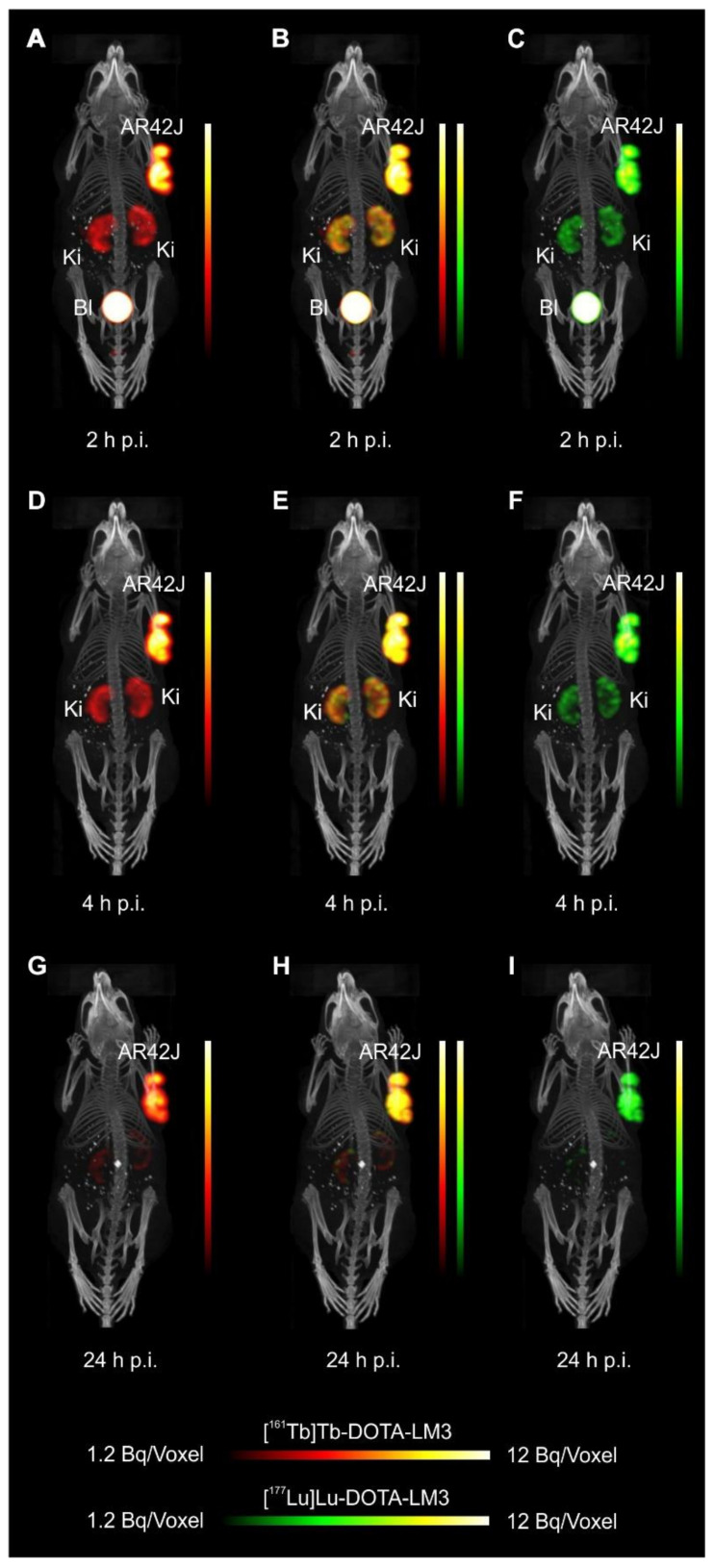
Dual-isotope SPECT/CT images of AR42J tumor-bearing mice shown as MIPs 2 h, 4 h and 24 h after injection of [^161^Tb]Tb-DOTA-LM3 (15 MBq, 0.5 nmol/mouse) and [^177^Lu]Lu-DOTA-LM3 (15 MBq, 0.5 nmol/mouse). (**A**–**C**) Scans acquired 2 h p.i. of the radiopeptides; (**D**–**F**) Scans acquired 4 h p.i. of the radiopeptides and (**G**–**I**) Scans acquired 24 h p.i. of the radiopeptides. (**A**,**D**,**G**) Reconstructions based on the X-rays and γ-lines of terbium-161; (**B**,**E**,**H**) Reconstructions based on the X-rays or γ-lines of terbium-161 and on the γ-lines lutetium-177; (**C**,**F**,**I**) Reconstructions based on the γ-lines of lutetium-177. AR42J = SSTR-positive tumor xenograft; Ki = kidney; Bl = urinary bladder.

The SPECT/CT images of AR42J tumor-bearing mice demonstrated equal in vivo distribution of simultaneously injected [^161^Tb]Tb-DOTATOC and [^177^Lu]Lu-DOTATOC (Figure 3). The same observation was made for [^161^Tb]Tb-DOTA-LM3 and [^177^Lu]Lu-DOTA-LM3 (Figure 4). Images reconstructed using the energies of either radiolanthanide (red-to-yellow scale and green-to-yellow scale for terbium-161 and lutetium-177, respectively), provided the distribution for each radiopeptide separately in the same mouse (Figure 3 and Figure 4). Experiments performed by co-injection of excess unlabeled peptide resulted in SSTR blockade and, hence, accumulation of the radiopeptides in AR42J tumors was not observed (Supplementary Materials, Figures S6 and S7). These additional studies proved that the uptake of the SST analogues in AR42J tumor xenografts was SSTR-specific.

Quantification of the accumulated activity in AR42J tumors and kidneys based on SPECT scans confirmed equal distribution of the ^161^Tb- and ^177^Lu-labeled counterparts (Supplementary Materials, Figure S8). This was the ultimate proof that the chosen radio-lanthanide (terbium-161 or lutetium-177) did not have an impact on the tissue distribution profile of the radiopeptides.

The SPECT/CT images showed activity accumulation in the AR42J xenografts, which was higher for the antagonist than for the agonist (Figure 3 and Figure 4). In agreement with quantitative data from biodistribution studies, the activity was efficiently cleared through the kidneys over time and almost entirely excreted after 24 h. Due to the favorable uptake of radiolabeled DOTA-LM3 in the tumor tissue, the tumor-to-kidney ratio was higher as compared to the ratio obtained after injection of radiolabeled DOTATOC.

### 3.7. Simultaneous Imaging Demonstrates Equal Sub-Organ Distribution of ^161^Tb- and ^177^Lu-Labeled SST Analogues

**Figure 5:**
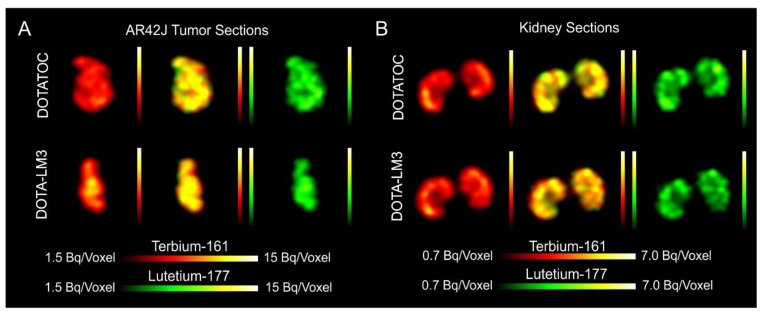
Dual-isotope SPECT images shown as coronal sections of AR42J tumors and kidneys. (**A**) Images of AR42J-tumors and (**B**) Images of the kidneys of mice 2 h p.i. of the radiopeptides. Mice were injected with a mixture of [^161^Tb]Tb-DOTATOC and [^177^Lu]Lu-DOTATOC (upper panel) or a mixture of [^161^Tb]Tb-DOTA-LM3 and [^177^Lu]Lu-DOTA-LM3 (lower panel). Reconstructions based on X-rays or γ-rays of terbium-161 (left); Reconstructions based on X-rays or γ-rays of terbium-161 and on the γ-rays of lutetium-177 (middle); reconstructions based on the γ-rays of lutetium-177 (right).

Dual-isotope SPECT image sections enabled, for the first time, visualization of the ^161^Tb- and ^177^Lu-labeled peptide distribution at a sub-organ level in the same animal. Most important to note is that the pattern of activity distribution in tumors and kidneys was the same, irrespective of whether terbium-161 or lutetium-177 was used. The uptake in the tumor was quite homogenous, which can be ascribed to the well-vascularized AR42J xenograft (Figure 5). Accumulation of activity in the kidneys was more prominent in the cortex where the megalin-mediated reabsorption of radiopeptides occurs [41], and where various SSTR subtypes are known to be expressed (Figure 5) [42,43].

## 4. Conclusions

In this study, we confirmed the previously postulated assumption that terbium-161 and lutetium-177 are interchangeable without altering the chemical and pharmacokinetic properties of the radiolabeled biomolecule. In dual-isotope SPECT/CT imaging studies, we demonstrated that the organ and sub-organ distribution of SST analogues labeled with terbium-161 and lutetium-177 was identical. These and previous findings suggest that any future (pre)clinical studies with terbium-161 can be based on preclinical data obtained with its ^177^Lu-labeled counterpart. This will allow the focusing of future investigations directly on the therapeutic efficacy of terbium-161, which is likely to be superior to the effect obtained with lutetium-177.

## Data Availability

The data presented in this study are available on request from the corresponding author.

## Supplementary Materials

The following data are available online at https://www.mdpi.com/article/10.3390/pharmaceutics13040536/s1. Figure S1: Chemical structures of the somatostatin (SST) analogues, Figure S2: Representative HPLC chromatograms of the ^161^Tb- and ^177^Lu-labeled peptides, Figure S3: Graphs of bars representing the percentage of intact radiopeptide investigated at 1 h, 4 h, and 24 h after preparation and dilution in saline (10 MBq/250 μL), Figure S4: In vitro AR42 tumor cell uptake of [^161^Tb]Tb-DOTA-LM3 after a 4 h incubation period in the absence and presence of excess DOTANOC or DOTA-LM3 as blocking agents (*n* = 2–3), Figure S5: SPECT/CT images shown as MIPs (upper pannel) and transaxial sections (lower pannel) of Vial 1 (V1) containing ~10 MBq of terbium-161, V2 containing ~10 MBq lutetium-177 and V3 with ~10 MBq terbium-161 and ~10 MBq lutetium-177, Figure S6: Dual-isotope SPECT/CT images of blocking studies carried out in AR42J tumor-bearing mice. Images are shown as maximum intensity projections (MIPs) 2 h and 4 h p.i. of [^161^Tb]Tb-DOTATOC (15 MBq, 0.5 nmol/mouse) and [^177^Lu]Lu-DOTATOC (15 MBq, 0.5 nmol/mouse) and excess unlabeled DOTATOC (20 nmol/mouse), Figure S7: Dual-isotope SPECT/CT images of blocking studies carried out in AR42J tumor-bearing mice. Images are shown as maximum intensity projections (MIPs) 2 h and 4 h p.i. of [^161^Tb]Tb-DOTA-LM3 (15 MBq, 0.5 nmol/mouse) and [^177^Lu]Lu-DOTA-LM3 (15 MBq, 0.5 nmol/mouse) and excess unlabeled DOTA-LM3 (20 nmol/mouse), Figure S8: Quantitative analysis of the SPECT images, Table S1: Biodistribution data obtained in AR42J tumor-bearing mice at 2 h and 24 h after injection of [^161^Tb]Tb-DOTATOC and [^177^Lu]Lu-DOTATOC, Table S2: Biodistribution data obtained in AR42J tumor-bearing mice at 2 h and 24 h after injection of [^161^Tb]Tb-DOTA-LM3 and [^177^Lu]Lu-DOTA-LM3.
