# Supplementary material for: Simultaneous Visualization of 161Tb- and 177Lu-Labeled Somatostatin Analogues Using Dual-Isotope SPECT Imaging
Source: Pharmaceutics. 2021 Apr 12;13(4):536. doi: 10.3390/pharmaceutics13040536 (PMC8070648; doi:10.3390/pharmaceutics13040536)
Supplement: Supplementary file 1 [file pharmaceutics-13-00536-s001.pdf]

pharmaceutics

MDPI

Supplementary Materials: Simultaneous Visualization of ^{161}Tb- and ^{177}Lu-Labeled Somatostatin Analogues Using Dual-Isotope SPECT Imaging

Francesca Borgna, Patrick Barritt, Pascal V. Grundler, Zeynep Talip, Susan Cohrs, Jan Rijn Zeevaart, Ulli Köster, Roger Schibli, Nicholas P. van der Meulen and Cristina Müller

S1. Somatostatin Analogues

Characteristics: DOTATOC and DOTA-LM3 are somatostatin (SST) analogues for targeting the somatostatin receptor (SSTR), as previously described in the literature (Figure S1) [1,2]. DOTATOC is an SSTR agonist while DOTA-LM3 acts as a SSTR antagonist. [^{177}Lu]Lu-DOTATOC is currently used in clinics for peptide receptor radionuclide therapy (PRRT) as a standard treatment of neuroendocrine tumors [3,4]. On the other hand, [^{177}Lu]Lu-DOTA-LM3 was only used in first-in-human studies, as reported in recent literature [5,6].

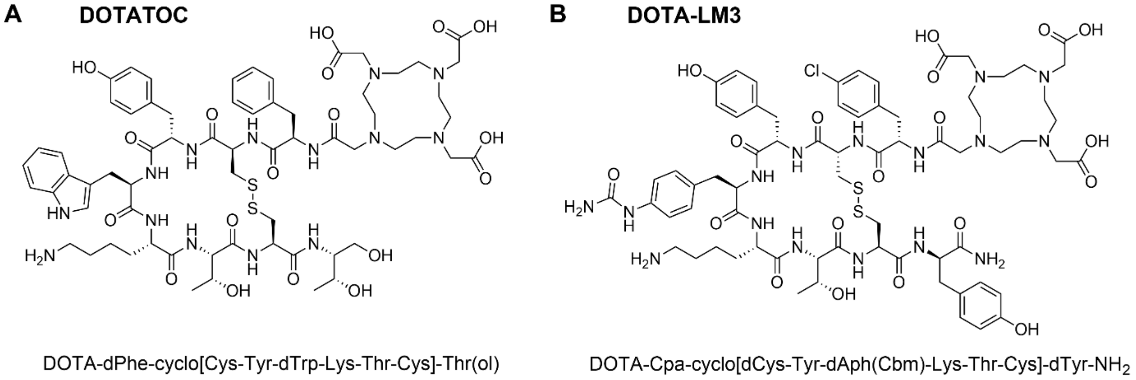

Figure S1. Chemical structures of the somatostatin (SST)analogues. (**A**) DOTATOC (somatostatin receptor (SSTR) agonist); (**B**) DOTA-LM3, (SSTR antagonist).

S2. Radiolabeling and Quality Control

Purpose: The SST analogues were labeled with terbium-161 and lutetium-177 to perform in vitro and in vivo experiments.

Methods: The stock solutions of DOTATOC and DOTA-LM3 were prepared in Milli-Q water to obtain a final concentration of 1 mM and stored at −20 °C. The SST analogues were labeled with terbium-161 or lutetium-177 using a 1:5 (*v*/*v*) mixture of sodium acetate (0.5 M) and HCl (0.05 M) at pH ~4.5 at a molar activity up to 100 MBq/nmol, according to a previously reported procedure [7-9]. The reaction mixture was incubated for 10 min at 95 °C. An aliquot of the radiopeptide labeling solution was diluted in Milli-Q water containing disodium diethylenetriamine pentaacetic acid (50 µM) for quality control using High Performance Liquid Chromatography (HPLC). A Merck Hitachi LaChrom HPLC system, equipped with a D-7000 interface, a L-7200 autosampler, a radioactivity detector (LB 506 B; Berthold Technologies GmbH & Co. KG, Bad Wildbad, Germany) and a L-7100 pump connected with a reversed-phase C18 column (Xterra™ MS, C18, 5 µm, 150 x 4.6 mm; Waters) was used to determine the radiochemical purity of the radiopeptides. The mobile phase consisted of 0.1% (*v*/*v*) TFA in Milli-Q water (A) and acetonitrile (B). A linear gradient of solution A (95–20%) and solvent B (5–80%) over 15 min was used at a flow rate of 1.0 mL/minute.

Results*:* The HPLC chromatograms of the quality control revealed high radiochemical purity (≥98%) of the ^{161}Tb- and ^{177}Lu-labeled SST analogues at a molar activity up to 100 MBq/nmol (Figure S2).

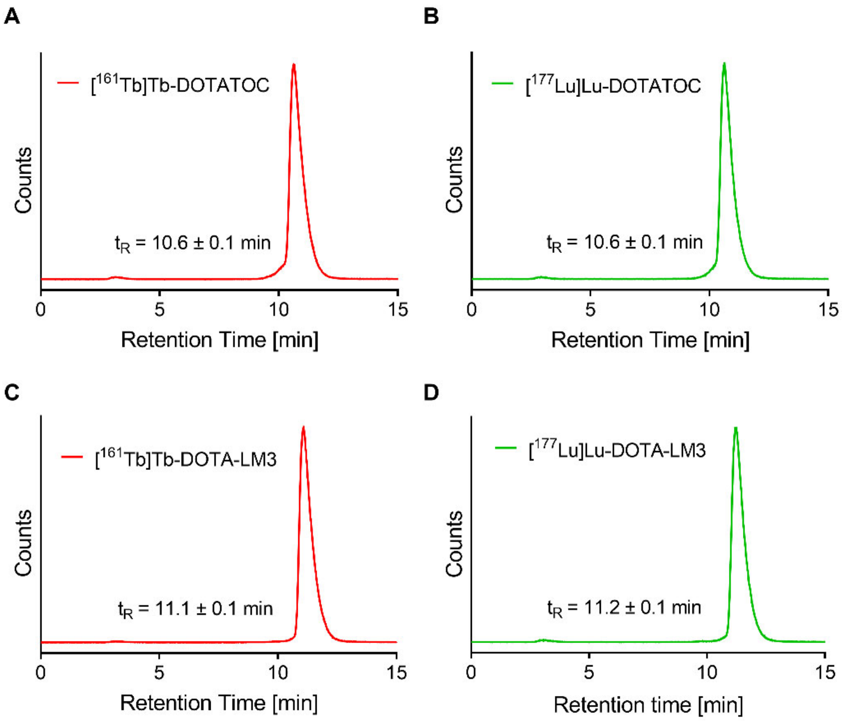

Figure S2. Representative HPLC chromatograms of the ^{161}Tb- and ^{177}Lu-labeled peptides. (**A**) [^{161}Tb]Tb-DOTATOC; (**B**) [^{177}Lu]Lu-DOTATOC; (**C**) [^{161}Tb]Tb-DOTA-LM3; (**D**) [^{177}Lu]Lu-DOTA-LM3. Traces of unreacted terbium-161 or lutetium-177 appeared with a retention time of ~2.5 min.

S3. Radiolytic Stability of the Radiopeptides

Purpose: The stability of the radiolabeled SST analogues was tested to confirm their integrity during in vitro and in vivo evaluation.

Methods: Radiolytic stability of the radiolabeled SST analogues was assessed over a period of 24 h (n = 2). For this purpose, DOTATOC and DOTA-LM3 were labeled with terbium-161 or lutetium-177 at a molar activity of 50 MBq/nmol. After quality control using HPLC (t = 0, radiochemical purity ≥98%, set as 100%), the labeling solutions were diluted with saline to a final volume of 250 µL at an activity concentration of 40 MBq/mL and incubated at room temperature with and without addition of L-ascorbic acid (120 µg/10 MBq in the final volume of 250 µL). Radiopeptide degradation was determined after 1 h, 4 h, and 24 h using HPLC. A quantitative assessment of the chromatograms was performed by expressing the integrated peak area of the intact product as the percentage of the sum of integrated peak areas of the entire chromatogram comprising released terbium-161 and lutetium-177, as well as degradation products of an unknown structure.

Results*:* The main findings are described in the main article and shown in Figure S3.

If the radiopeptides were used at concentrations below 40 MBq/mL and/or immediately after preparation, the addition of L-ascorbic acid was not necessary. Dual-isotope single photon emission computed tomography (SPECT) imaging studies were performed with activity concentrations above 40 MBq/mL (>4 MBq/100 µL/mouse), which required the addition of L-ascorbic acid (~300 µg/30 MBq/100 µL) to ensure integrity of the radiopeptides.

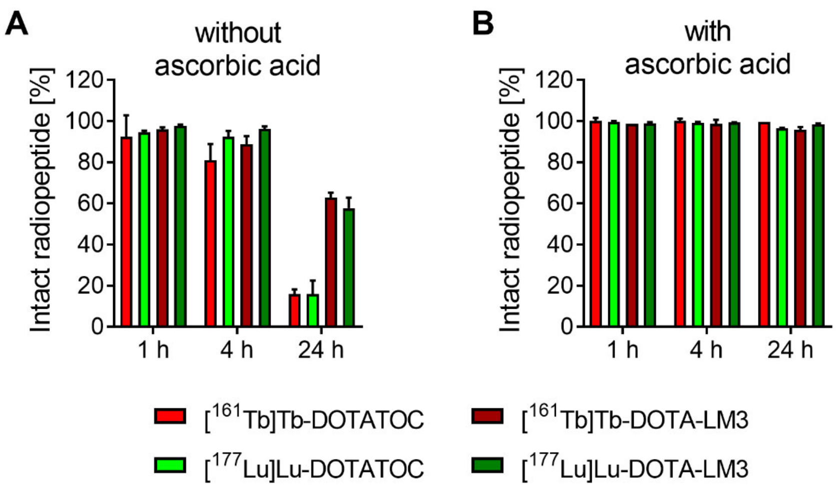

Figure S3. Graphs of bars representing the percentage of intact radiopeptide investigated at 1 h, 4 h, and 24 h after preparation and dilution in saline (10 MBq/250 µL). (**A**) Percentage of intact radiopeptide in solution in the absence of ascorbic acid; (**B**) Percentage of intact radiopeptide in solution in the presence of ascorbic acid.

S4. Determination of *n*-Octanol/PBS Distribution Coefficients (LogD Values)

Purpose: The *n*-octanol/PBS distribution coefficients (logD values) were assessed for each radiopeptide in order to investigate their hydrophilicity and enable comparison of the two analogues.

Methods: The logD values of the radiolabeled SST analogues (DOTATOC and DOTA-LM3) were determined by a shake-flask method, as previously reported [10]. The SST analogues were labeled with terbium-161 or lutetium-177 at a molar activity of 30 MBq/nmol. An aliquot of the radiolabeled peptide (0.5 MBq, 25 µL) was added to a mixture of 1475 µL PBS pH 7.4 and 1500 µL *n*-octanol. The respective tubes were vortexed for 60 s and centrifuged for 6 min at 2500 rpm followed by the measurement of the activity concentration in a defined volume of each layer using a γ-counter (Wallac Wizard 1480, Perkin Elmer Waltham, MA, USA). The logD values were calculated as the logarithm of the ratio of counts per minute (cpm) measured in the *n*-octanol phase relative to the cpm measured in the PBS pH 7.4 phase. The experiments were performed three times with five replicates for each radiopeptide and the logD value was expressed as the average ± standard deviation (SD) of the values obtained in each experiment. The data were analyzed for significance using a two-way ANOVA with Tukey's multiple comparisons test with GraphPad Prism (version 8.0, Graph Pad software, San Diego, CA, USA). A *p*-value <0.05 was considered statistically significant.

Results*:* The results are reported in the main article.

S5. Specificity of the Cellular Uptake of [^{161}Tb]Tb-DOTA-LM3

Purpose: In order to demonstrate that the uptake of [^{161}Tb]Tb-DOTA-LM3 is SSTR-specific, blocking studies were performed with excess unlabeled DOTANOC and DOTA-LM3, respectively.

Methods: A potential blockade of the uptake of [^{161}Tb]Tb-DOTA-LM3 into AR42J cells was determined in the presence of >1000-fold excess unlabeled DOTANOC or DOTA-LM3 relative to the radiopeptide using the same protocol as reported in the main article. AR42J cells were incubated with DOTANOC or DOTA-LM3 30–40 min before the addition of the radiopeptide.

Results: The addition of excess unlabeled DOTANOC to AR42J cells partially blocked the uptake of [^{161}Tb]Tb-DOTA-LM3 (from 64 ± 5% to 18 ± 1% of added activity)

after an incubation period of 4 h (Figure S4). The addition of excess unlabeled DOTA-LM3 reduced the uptake much more effectively, resulting in <0.3% [^{161}Tb]Tb-DOTA-LM3 associated with the cells. These results are discussed in the main article.

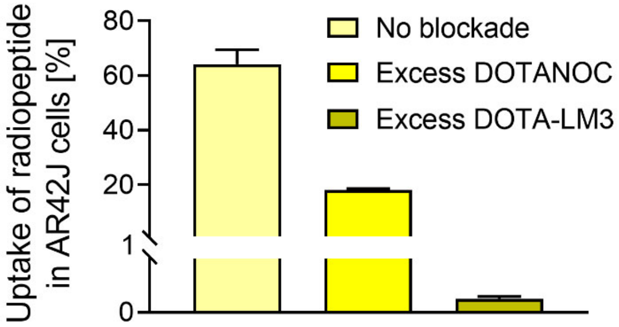

Figure S4. In vitro AR42 tumor cell uptake of [^{161}Tb]Tb-DOTA-LM3 after a 4 h incubation period in the absence and presence of excess DOTANOC or DOTA-LM3 as blocking agents (n = 2–3).

S6. Dual-Isotope SPECT/CT Imaging: Evaluation of the Crosstalk

Purpose: A dual-isotope SPECT protocol for terbium-161 and lutetium-177 was set up and validated using test vials filled with either radionuclide or both together to evaluate a potential crosstalk between terbium-161 and lutetium-177.

Methods: SPECT/CT scans were performed with a 4-head multi-plexing, multi-pinhole, small-animal SPECT/CT scanner (NanoSPECT/CT™, Mediso Medical Imaging Systems, Budapest, Hungary). Each head was outfitted with a tungsten-based aperture of nine 1.4 mm-diameter pinholes and a thickness of 10 mm (mouse whole body standard aperture (NSP-108-M14-WB) [11]). The scans were acquired using Nucline software (version 1.02, Mediso Ltd., Budapest, Hungary). Simultaneous acquisition of terbium-161 and lutetium-177 was enabled by detection of the photon emission of terbium-161 and lutetium-177. SPECT data were reconstructed iteratively using HiSPECT software (version 1.4.3049, Scivis GmbH, Göttingen, Germany) based on the respective energy windows for terbium-161 and lutetium-177 as reported in the main article. SPECT and CT data were automatically co-registered, as both modalities shared the same axis of rotation.

To evaluate a potential crosstalk of terbium-161 and lutetium-177, three 1-mL Eppendorf tubes (V1-V3) were filled each with either ~10 MBq terbium-161 (exact measurement: 9.7 MBq, 1.0 mL; V1), ~10 MBq lutetium-177 (exact measurement: 11.9 MBq, 1.0 mL; V2) or a mixture of ~10 MBq terbium-161 and ~10 MBq lutetium-177 in 1.0 mL (V3), respectively. All three vials were scanned simultaneously with a frame time of 20 s using the dual-isotope SPECT imaging protocol. The concentration of terbium-161 and lutetium-177 in each vial was determined using the quantification tool of the VivoQuant postprocessing software (version 3.5, inviCRO Imaging Services and Sofware, Boston, USA). Three regions of interest were selected in each vial and the average activity concentration ± SD of each radionuclide (kBq/mm^3) was calculated. The activity concentration of terbium-161 was compared between V1 and V3, while the activity concentration of lutetium-177 was compared between V2 and V3.

Results: The images of the vials shown as maximum intensity projections (MIPs) and transaxial sections of V1, V2, and V3 revealed no interference between terbium-161 and lutetium-177 in the acquired scans (Figure S5). Each radionuclide was visualized independently of the other with high accuracy.

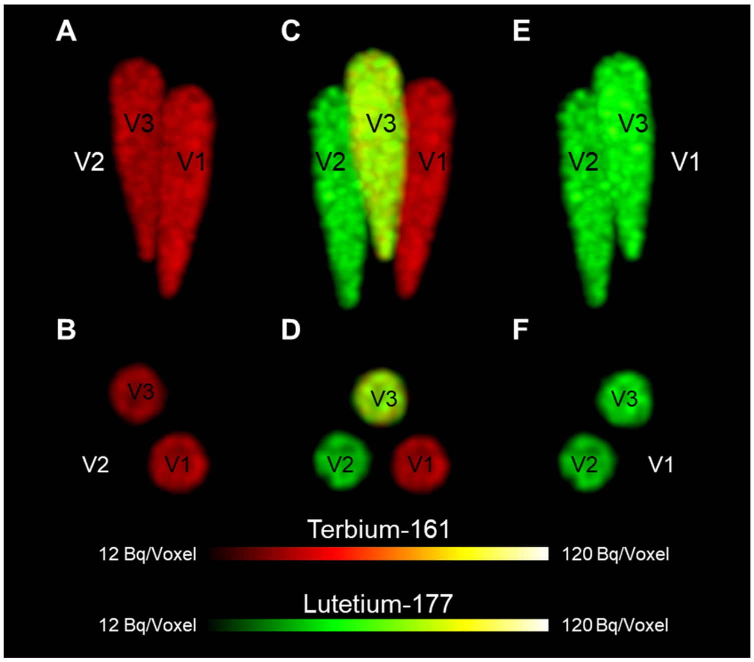

Figure S5. SPECT/CT images shown as MIPs (upper pannel) and transaxial sections (lower pannel) of Vial 1 (V1) containing ~10 MBq of terbium-161, V2 containing ~10 MBq lutetium-177 and V3 with ~10 MBq terbium-161 and ~10 MBq lutetium-177. (**A**/**B**) Reconstructions based on X-rays or γ-rays of terbium-161; (**C**/**D**) Reconstructions based on X-rays or γ-rays of terbium-161 and on the γ-rays of lutetium-177; (**E**/**F**) Reconstructions based on the γ-rays of lutetium-177.

The determined concentration of terbium-161 was 11.5 ± 0.7 kBq/mm^3 and 10.2 ± 0.6 kBq/mm^3 in V1 and V3, respectively. The activity concentration of lutetium-177 was 12.4 ± 0.7 kBq/mm^3 and 13.7 ± 0.7 kBq/mm^3 in V2 and V3, respectively. Importantly, no activity stemming from terbium-161 was detected in the lutetium-177 vial (V2) (<0.01 kBq/mm^3) and no activity stemming from lutetium-177 was detected in the terbium-161 vial (V1) (<0.02 kBq/mm^3).

S7. Dual-Isotope SPECT/CT Imaging: In Vivo Studies

Purpose: The protocol was used for the simultaneous visualization of the distribution profile of ^{161}Tb- and ^{177}Lu-labeled DOTATOC or DOTA-LM3 in mice. The activity accumulation in tumors and kidneys was quantified based on the SPECT scans.

Methods: *SPECT/CT Imaging Studies:* The imaging studies with AR42J tumor-bearing mice were performed with either a mixture of ^{161}Tb- and ^{177}Lu-labeled DOTATOC or with a mixture of ^{161}Tb- and ^{177}Lu-labeled DOTA-LM3 at a terbium-161/lutetium-177 activity ratio of 1:1, as described in the main article. SPECT/CT scans were acquired 2 h, 4 h and 24 h after injection of the radiopeptides using the established dual-isotope SPECT acquisition protocol. Blocking experiments were performed 2 h and 4 h after injection of the radiopeptides that were prepared and mixed as described in the main article; however, an excess (20 nmol/mouse) of the respective unlabeled peptide was added to the injection solution. The mice were anaesthetized using a mixture of isoflurane and oxygen. The image processing of the dataset is described in the main article.

Quantification of Accumulated Activity in Tumors and Kidneys: The reconstructed SPECT scans (n = 3 per radiopeptide) were used to quantify the accumulation of [^{161}Tb]Tb-DOTATOC and [^{177}Lu]Lu-DOTATOC or [^{161}Tb]Tb-DOTA-LM3 and [^{177}Lu]Lu-DOTA-LM3 in tumors and kidneys of each mouse. The activity in these tissues was also quantified in mice that received the radiopeptides with an excess of the unlabeled peptide (n = 2). The quantification tool of the VivoQuant postprocessing software (version 3.5, inviCRO

Imaging Services and Sofware, Boston, USA) was used to allow defining a region of interest around the respective organ or tissue. The results were presented as percentage of injected activity per tumor (% IA/tumor) or per kidney (% IA/kidney), respectively. The results were presented as the average ± SD. Statistical differences between ^{161}Tb- and ^{177}Lu-labeled SST analogues were calculated with a two-way ANOVA using a Tukey's multi comparisons post-test.

Results: The dual-isotope SPECT/CT experiments are shown in the main article. The scans of the mice which received a co-injection of the radiopeptide with an excess of unlabeled peptide showed no accumulation of activity in AR42J tumors, proving that the tumor uptake of both radiopeptides was SSTR-specific (Figure S6/S7 and Figure S8).

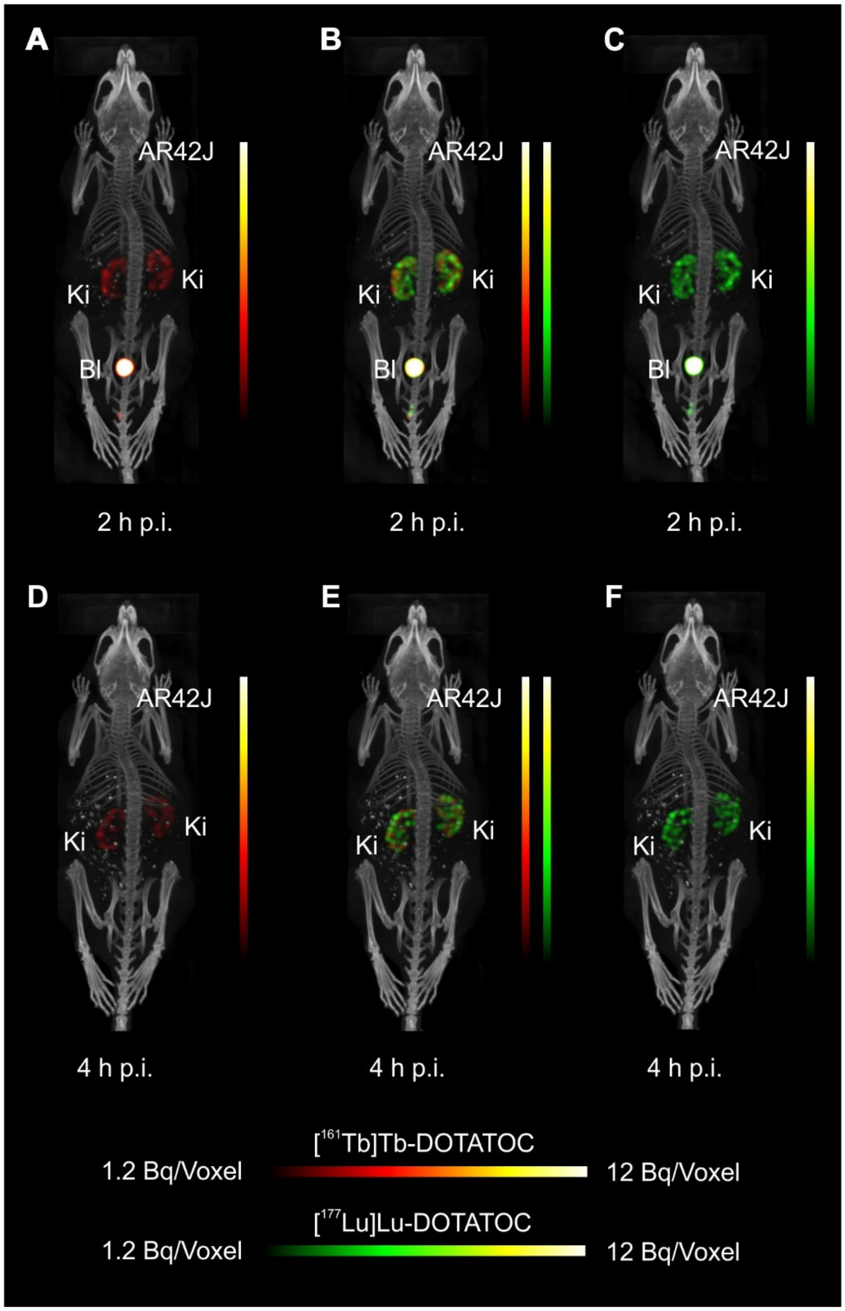

Figure S6. Dual-isotope SPECT/CT images of blocking studies carried out in AR42J tumor-bearing mice. Images are shown as maximum intensity projections (MIPs) 2 h and 4 h p.i. of [^{161}Tb]Tb-DOTATOC (15 MBq, 0.5 nmol/mouse) and [^{177}Lu]Lu-DOTATOC (15 MBq, 0.5 nmol/mouse) and excess unlabeled DOTATOC (20 nmol/mouse). (**A**/**B**/**C**) Scans acquired 2 h p.i. of the radiopeptides; (**D**/**E**/**F**) Scans acquired 4 h p.i. of the radiopeptides. (**A**/**D**) Reconstructions based on the X-rays and γ-lines of terbium-161; **(B/E)** Reconstructions based on the X-rays and γ-lines of terbium-161 and the γ-lines of lutetium-177; **(C/F)** Reconstructions based on the γ-lines of lutetium-177. AR42J = SSTR-positive tumor xenograft; Ki = kidney; Bl = urinary bladder.

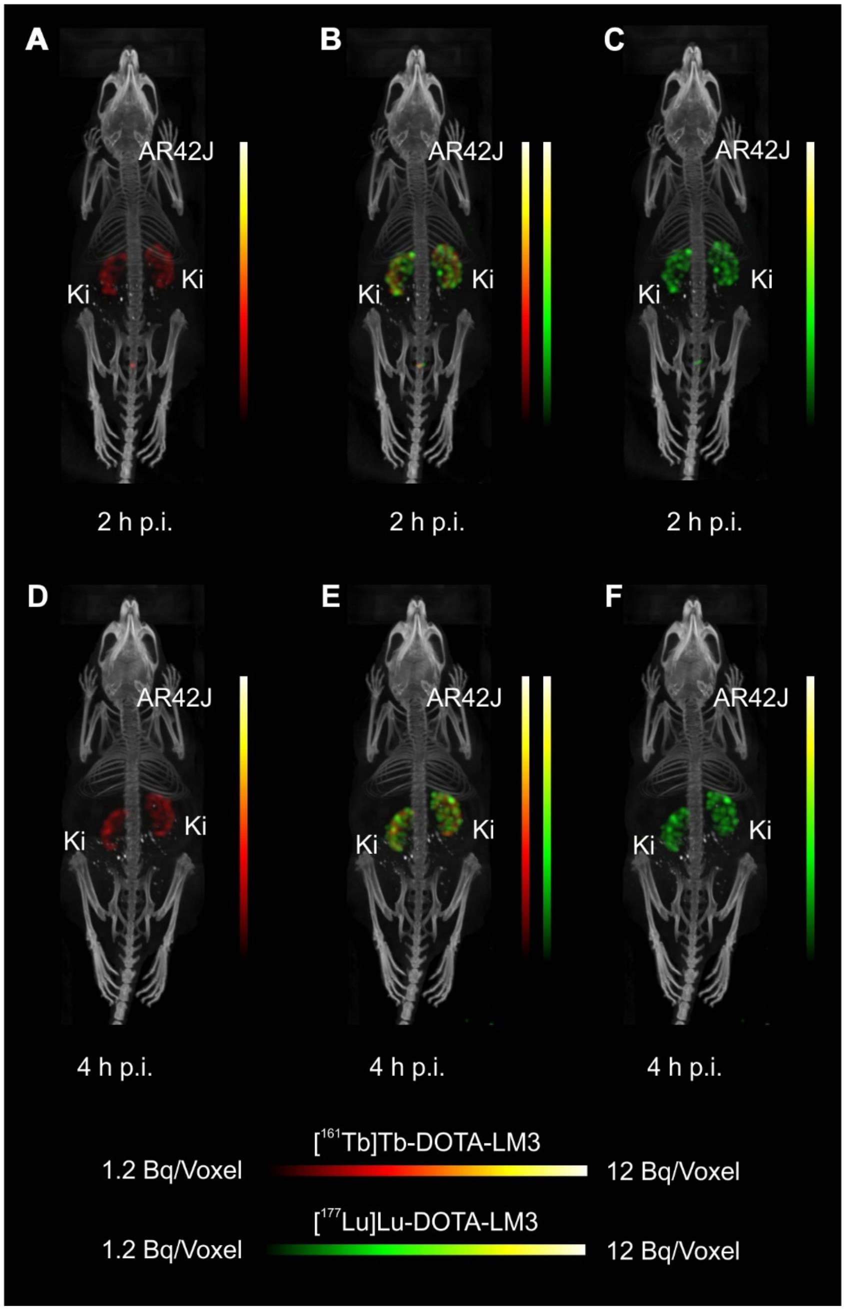

Figure S7. Dual-isotope SPECT/CT images of blocking studies carried out in AR42J tumor-bearing mice. Images are shown as maximum intensity projections (MIPs) 2 h and 4 h p.i. of [^{161}Tb]Tb-DOTA-LM3 (15 MBq, 0.5 nmol/mouse) and [^{177}Lu]Lu-DOTA-LM3 (15 MBq, 0.5 nmol/mouse) and excess unlabeled DOTA-LM3 (20 nmol/mouse). (**A**/**B**/**C**) Scans acquired 2 h p.i. of the radiopeptides; (**D**/**E**/**F**) Scans acquired 4 h p.i. of the radiopeptides. (**A**/**D**) Reconstructions based on the X-rays and γ-lines of terbium-161; **(B/E)** Reconstructions based on the X-rays and γ-lines of terbium-161 and the γ-lines of lutetium-177; **(C/F)** Reconstructions based on the γ-lines of lutetium-177. AR42J = SSTR-positive tumor xenograft; Ki = kidney; Bl = urinary bladder.

The quantification of the accumulated activity in AR42J tumors and in kidneys confirmed equal distribution profiles of the ^{161}Tb- and ^{177}Lu-labeled counterparts ($p > 0.05$), as already demonstrated by quantitative biodistribution data.

Moreover, it was shown that the accumulated activity in AR42J tumor xenografts was dramatically reduced in mice that received a co-injection of excess cold peptide ($p < 0.05$).

These findings confirm SSTR-specific tumor uptake of the radiopeptides. Retention of activity in kidneys was, however, only partially reduced in these mice due to the fact that renal uptake of SST analogues is only partially mediated by SSTRs but mainly due to the megalin-mediated reabsorption [12], as described in the main article (Figure S8).

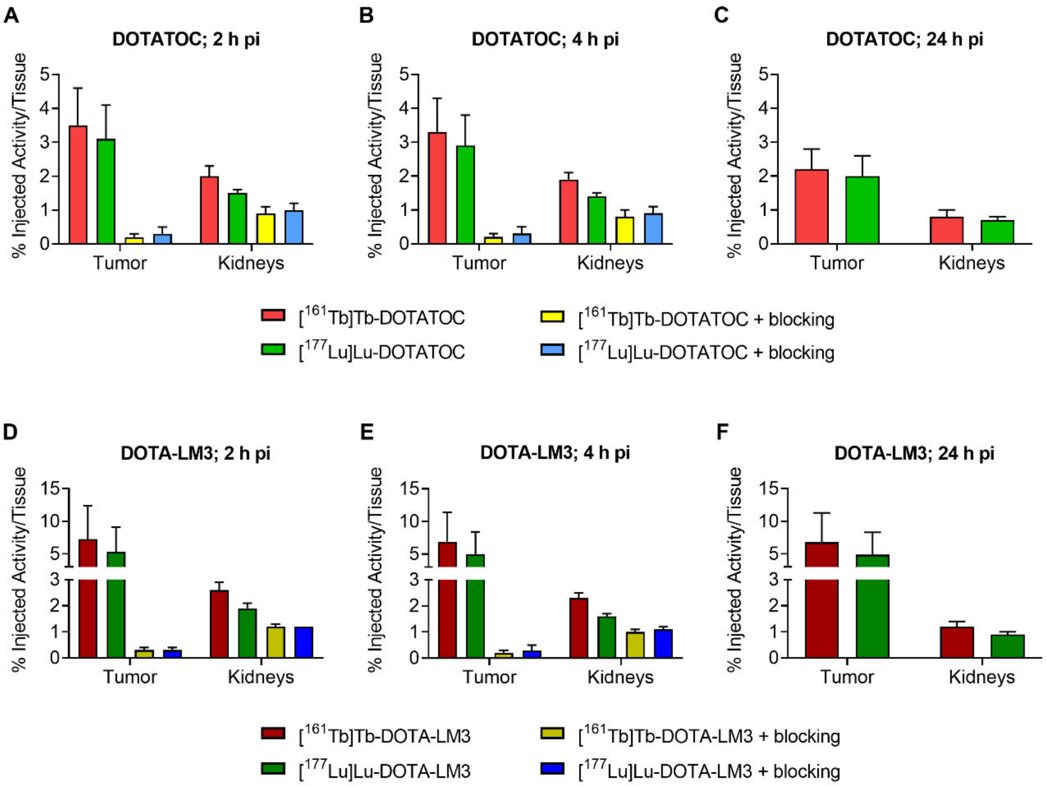

Figure S8. Quantitative analysis of the SPECT images. The uptake was expressed as percentage of injected activity per tumor or kidneys, respectively. (**A**/**B**/**C**) Uptake of [^{161}Tb]Tb-DOTATOC and [^{177}Lu]Lu-DOTATOC in the AR42J xenograft (tumor) and kidneys at 2 h p.i. (**A**), at 4 h p.i. and (**B**) at 24 h p.i. (**C**). (**D**/**E**/**F**) Uptake of [^{161}Tb]Tb-DOTA-LM3 and [^{177}Lu]Lu-DOTA-LM3 in the tumor and kidneys at 2 h p.i. (**D**), at 4 h p.i. and (**E**) at 24 h p.i. (**F**). Each mouse was injected with a mixture of [^{161}Tb]Tb-DOTATOC (15 MBq; 0.5 nmol/mouse) and [^{177}Lu]Lu-DOTATOC (15 MBq; 0.5 nmol/mouse) or [^{161}Tb]Tb-DOTA-LM3 (15 MBq; 0.5 nmol/mouse) and [^{177}Lu]Lu-DOTA-LM3 (15 MBq; 0.5 nmol/mouse) (n = 3). Blocking studies were performed by co-injection of excess (20 nmol/mouse) of the respective unlabeled peptide (n = 2).

S8. Biodistribution Studies: Comparison of ^{161}Tb- and ^{177}Lu-labeled Peptides

Purpose: Biodistribution studies were performed with ^{161}Tb- and ^{177}Lu-labeled DOTATOC and DOTA-LM3 to compare the distribution profile of the two radiopeptides and to confirm quantitatively that the ^{161}Tb-and ^{177}Lu-labeled counterparts show the same tissue distribution.

Methods: The methods are reported in the main article.

Results: The results were decay-corrected and listed as percentage of the injected activity per gram of tissue mass (% IA/g). They are described and discussed in the main article and reported in Tables S1/S2.

Table S1. Biodistribution data obtained in AR42J tumor-bearing mice at 2 h and 24 h after injection of [^{161}Tb]Tb-DOTATOC and [^{177}Lu]Lu-DOTATOC, respectively. Decay-corrected data are shown as [% IA/g] values, representing the average ± SD (*n* = 3–4).

Radiopeptide	[^{161}Tb]Tb-DOTATOC		[^{177}Lu]Lu-DOTATOC	
**Tissue**	**2 h p.i.**	**24 h p.i.**	**2 h p.i.**	**24 h p.i.**
Blood	≤0.10	≤0.10	≤0.10	≤0.10
Heart	≤0.10	≤0.10	≤0.10	≤0.10
Lung	0.27 ± 0.03	0.11 ± 0.01	0.37 ± 0.06	0.12 ± 0.03
Spleen	0.11 ± 0.02	≤0.10	0.15 ± 0.03	≤0.10
Kidneys	9.9 ± 1.4	5.2 ± 0.6	11 ± 1	5.9 ± 2.1
Adrenals	0.25 ± 0.06	0.23 ± 0.20	0.28 ± 0.08	0.18 ± 0.08
Stomach	0.74 ± 0.38	0.29 ± 0.11	0.65 ± 0.11	0.24 ± 0.04
Pancreas	0.71 ± 0.07	0.18 ± 0.01	0.80 ± 0.13	0.21 ± 0.04
Intestines	0.21 ± 0.13	≤0.10	0.20 ± 0.05	≤0.10
Liver	0.17 ± 0.01	≤0.10	0.26 ± 0.07	0.13 ± 0.02
Muscle	≤0.10	≤0.10	≤0.10	≤0.10
Femur	≤0.10	≤0.10	≤0.10	≤0.10
AR42J Tumor	8.2 ± 0.2	3.8 ± 0.6	8.9 ± 1.5	3.8 ± 0.3
Tumor-to-blood	260 ± 13	433 ± 71	146 ± 16	456 ± 118
Tumor-to-liver	49 ± 1	41 ± 7	36 ± 7	31 ± 6
Tumor-to-kidney	0.84 ± 0.12	0.73 ± 0.10	0.73 ± 0.07	0.72 ± 0.34

Table S2. Biodistribution data obtained in AR42J tumor-bearing mice at 2 h and 24 h after injection of [^{161}Tb]Tb-DOTA-LM3 and [^{177}Lu]Lu-DOTA-LM3, respectively. Decay-corrected data are shown as [% IA/g] values, representing the average ± SD (*n* = 3–4).

Radiopeptide	[^{161}Tb]Tb-DOTA-LM3		[^{177}Lu]Lu-DOTA-LM3	
**Tissue**	**2 h p.i.**	**24 h p.i.**	**2 h p.i.**	**24 h p.i.**
Blood	≤0.10	≤0.10	≤0.10	≤0.10
Heart	0.11 ± 0.02	≤0.10	≤0.10	≤0.10
Lung	1.0 ± 0.2	0.36 ± 0.06	0.83 ± 0.14	0.28 ± 0.02
Spleen	0.27 ± 0.06	0.13 ± 0.03	0.23 ± 0.06	≤0.10
Kidneys	12 ± 1	5.8 ± 0.1	11 ± 1	5.4 ± 1.3
Adrenals	0.44 ± 0.06	0.18 ± 0.05	0.37 ± 0.10	0.17 ± 0.04
Stomach	2.2 ± 0.3	0.69 ± 0.14	1.7 ± 0.4	0.86 ± 0.13
Pancreas	4.2 ± 0.3	1.9 ± 0.1	3.4 ± 0.3	1.5 ± 0.1
Intestines	0.43 ± 0.23	0.11 ± 0.01	0.31 ± 0.14	0.14 ± 0.03
Liver	0.45 ± 0.05	0.22 ± 0.05	0.35 ± 0.08	0.15 ± 0.03
Muscle	≤0.10	≤0.10	≤0.10	≤0.10
Femur	0.20 ± 0.05	0.11 ± 0.02	0.17 ± 0.04	≤0.10
AR42J Tumor	18 ± 2	14 ± 2	17 ± 2	14 ± 2
Tumor-to-blood	200 ± 38	>1000	270 ± 92	>1000
Tumor-to-liver	38 ± 7	63 ± 18	51 ± 7	95 ± 16
Tumor-to-kidney	1.4 ± 0.2	2.4 ± 0.5	1.9 ± 0.2	2.9 ± 0.9

References

1. Fani, M.; Braun, F.; Waser, B.; Beetschen, K.; Cescato, R.; Erchegyi, J.; Rivier, J.E.; Weber, W.A.; Maecke, H.R.; Reubi, J.C. Unexpected sensitivity of SST2 antagonists to N-terminal radiometal modifications. *J. Nucl. Med.* **2012**, *53*, 1481–1489, doi:10.2967/jnumed.112.102764.
2. Fani, M.; Del Pozzo, L.; Abiraj, K.; Mansi, R.; Tamma, M.L.; Cescato, R.; Waser, B.; Weber, W.A.; Reubi, J.C.; Maecke, H.R. PET of somatostatin receptor-positive tumors using ^{64}Cu- and ^{68}Ga-somatostatin antagonists: the chelate makes the difference. *J. Nucl. Med.* **2011**, *52*, 1110–1118, doi:10.2967/jnumed.111.087999.

3. Baum, R.P.; Kluge, A.W.; Kulkarni, H.; Schorr-Neufing, U.; Niepsch, K.; Bitterlich, N.; van Echteld, C.J. [^{177}Lu-DOTA]^{0}-D-Phe^{1}-Tyr^{3}-octreotide (^{177}Lu-DOTATOC) for peptide receptor radiotherapy in patients with advanced neuroendocrine tumours: a Phase-II study. *Theranostics* **2016**, *6*, 501–510, doi:10.7150/thno.13702.
4. Wang, L.F.; Lin, L.; Wang, M.J.; Li, Y. The therapeutic efficacy of ^{177}Lu-DOTATATE/DOTATOC in advanced neuroendocrine tumors: A meta-analysis. *Medicine (Baltimore)* **2020**, *99*, e19304, doi:10.1097/MD.0000000000019304.
5. Zhang, J.; Kulkarni, H.R.; Singh, A.; Baum, R.P. Successful Intra-arterial peptide receptor radionuclide therapy of DOTATOC-negative high-grade liver metastases of a pancreatic neuroendocrine neoplasm using ^{177}Lu-DOTA-LM3: a somatostatin receptor antagonist. *Clin. Nucl. Med.* **2020**, *45*, e165–e168, doi:10.1097/RLU.0000000000002906.
6. Baum, R.P.; Zhang, J.; Schuchardt, C.; Müller, D.; Mäcke, H. First-in-human study of novel SSTR antagonist ^{177}Lu-DOTA-LM3 for peptide receptor radionuclide therapy in patients with metastatic neuroendocrine neoplasms: dosimetry, safety and efficacy. *J. Nucl. Med.* **2021**, 10.2967/jnumed.120.258889, doi:10.2967/jnumed.120.258889.
7. Müller, C.; Reber, J.; Haller, S.; Dorrer, H.; Bernhardt, P.; Zhernosekov, K.; Türler, A.; Schibli, R. Direct in vitro and in vivo comparison of ^{161}Tb and ^{177}Lu using a tumour-targeting folate conjugate. *Eur. J. Nucl. Med. Mol. Imaging* **2014**, *41*, 476–485, doi:10.1007/s00259-013-2563-z.
8. Haller, S.; Pellegrini, G.; Vermeulen, C.; van der Meulen, N.P.; Köster, U.; Bernhardt, P.; Schibli, R.; Müller, C. Contribution of Auger/conversion electrons to renal side effects after radionuclide therapy: preclinical comparison of ^{161}Tb-folate and ^{177}Lu-folate. *EJNMMI Res.* **2016**, *6*, 13, doi:10.1186/s13550-016-0171-1.
9. Müller, C.; Umbricht, C.A.; Gracheva, N.; Tschan, V.J.; Pellegrini, G.; Bernhardt, P.; Zeevaart, J.R.; Köster, U.; Schibli, R.; van der Meulen, N.P. Terbium-161 for PSMA-targeted radionuclide therapy of prostate cancer. *Eur. J. Nucl. Med. Mol. Imaging* **2019**, *46*, 1919–1930, doi:10.1007/s00259-019-04345-0.
10. Müller, C.; Mindt, T.L.; de Jong, M.; Schibli, R. Evaluation of a novel radiofolate in tumour-bearing mice: promising prospects for folate-based radionuclide therapy. *Eur. J. Nucl. Med. Mol. Imaging* **2009**, *36*, 938–946.
11. Schramm, N.; Hoppin, J.; Lackas, C.; Gershman, B.; Norenberg, J.; de Jong, M. Improving resolution, sensitivity and applications for the NanoSPECT/CT: a high-performance SPECT/CT imager for small-animal research. *J. Nucl. Med.* **2007**, *48 (suppl 2)*, 436P.
12. Vegt, E.; Melis, M.; Eek, A.; de Visser, M.; Brom, M.; Oyen, W.J.; Gotthardt, M.; de Jong, M.; Boerman, O.C. Renal uptake of different radiolabelled peptides is mediated by megalin: SPECT and biodistribution studies in megalin-deficient mice. *Eur. J. Nucl. Med. Mol. Imaging* **2011**, *38*, 623–632, doi:10.1007/s00259-010-1685-9.
